# Retinal surgery quality indicators for uncomplicated primary rhegmatogenous retinal detachment without a national registry

**DOI:** 10.1111/aos.15138

**Published:** 2022-03-29

**Authors:** Jan‐Olof Carlsson, Otto Fricke, Anton Dahlberg, Sven Crafoord

**Affiliations:** ^1^ Department of Ophthalmology Örebro University Hospital Örebro Sweden; ^2^ Department of Ophthalmology Linköping University Linköping Sweden; ^3^ Faculty of Medicine and Health, Department of Ophthalmology Örebro University Örebro Sweden

**Keywords:** final success, postoperative endophthalmitis, primary anatomical success, retrospective

## Abstract

**Purpose:**

The objective of this study was to evaluate the possibility of analysing quality indicators for uncomplicated primary rhegmatogenous retinal detachment in a hospital department of ophthalmology without the support of a national registry or need to collect data from referring ophthalmological centres.

**Methods:**

In 2014, we operated 231 consecutive eyes with uncomplicated retinal detachment. Our quality indicators were primary anatomical success, final anatomical success and postoperative endophthalmitis. We reviewed medical records in our university surgical department retrospectively and compared them with medical records from the regional hospitals that had referred most of the operated patients and done their own postoperative examination. Our hypothesis was that any retinal re‐detachment and/or serious postoperative complication would be reported back.

**Results:**

The medical records at the surgical department revealed primary anatomic success for 91.3% of eyes and final anatomical success of 99.6%. The data from the regional hospitals confirmed that our hypothesis was correct. All patients with adverse outcomes were referred back for reoperation. Patients who were not referred again had an attached retina and showed no signs of endophthalmitis.

**Conclusion:**

Our hypothesis that data in the surgical department's medical records would closely reflect those in referring hospitals was borne out. This supports, under current conditions, an effective strategy for analysing chosen quality indicators without relying on a national registry or reviewing records from regional hospitals.

## Introduction

Primary rhegmatogenous retinal detachment is one of the most common eye diseases surgically treated at the vitreoretinal centre in Örebro University Hospital in Sweden. While some countries have national registries to follow up retinal detachment surgery (Hajari et al. 2015), Sweden has no such facility despite far‐reaching discussions and a previous national registry that ran for a few years in the 1990s (Algvere et al. 1999). With the lack of a national register, every vitreoretinal surgical unit in Sweden is responsible for acquiring its own postoperative information on its treated patients.

All seven university hospitals in Sweden have a vitreoretinal department capable of advanced surgery for its own patients and those referred from surrounding regional hospitals. The eye clinic at the university hospital in Örebro treats retinal detachment for both local patients and referrals from several ophthalmological centres in the middle region of Sweden (total population ∼ 2–3 million).

Thorough follow‐up of vitreoretinal surgery is necessary and desirable. The most obvious reason for this is to educate new surgeons and to keep them and more experienced surgeons up to date as new technologies develop. Follow‐up knowledge also facilitates the transfer of information to patients about their expected results and any potential complications. Politicians have been increasingly interested in ensuring that different regions have equal access to resources and demonstrate equal competence and quality of care, leading currently to both political and healthcare demands that surgical units publish or otherwise present their results.

In everyday clinical practice, it is not always possible or desirable to monitor operated patients for 6 months simply to meet the requirements for an academic report. All regional records in this study were reviewed at least 2 years after the primary operation. Patients who died before 6 months were excluded, and the date of the last visit was the end date for follow‐up. Had the patient not made new contact at the time of journal review, we assumed that the process was uncomplicated.

Our hypothesis in this study was that regional hospitals reliably notify the surgical unit or re‐refer patients who suffer a postoperative detached retina and/or other significant postoperative complications. If the hypothesis were true, the surgical unit charts would contain all relevant information about deviant recoveries and major complications for all patients, both local and referred from a regional hospital.

This would mean that if there was no other information in the medical journal at the surgical unit other than the discharge notes, we could assume the recovery developed as intended.

The purpose of this study was to review a simple method to continuously evaluate our chosen quality indicators in uncomplicated primary retinal detachment surgery (Hajari et al. 2015) for its effectiveness, ease of use, safety and reliability.

## Material and Method

In 2014, we performed 304 operations with the diagnosis of primary retinal detachment. At each operation, the surgeon registered the diagnosis. Based on the inclusion criteria, 231 surgeries were considered uncomplicated and eligible for inclusion. All postoperative data for this study came from medical records at the university hospital and the regional hospitals. Follow‐up was straightforward for patients connected to the surgical unit. Their medical records were easily accessible to the surgical department, unlike those from the regional hospitals, where safety issues and administrative demands tended to slow down and prolong the time required to collect data.The study was a retrospective and consecutive follow‐up of surgeries performed at the Eye Department, Örebro University Hospital, which serves its own area and several surrounding hospitals.


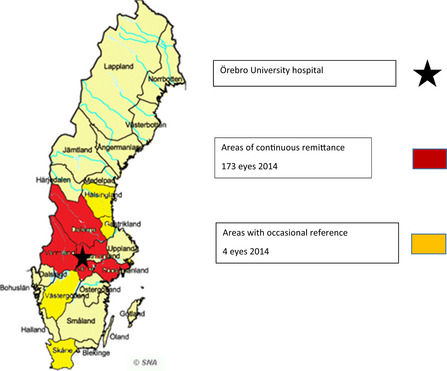


We chose primary success (anatomic success, one operation), final success (anatomic success, several operations) and postoperative endophthalmitis following the primary operation as our quality indicators. Follow‐up time is defined by the last complete retinal assessment.

*The inclusion criterion* for defining a condition as ‘uncomplicated’ was rhegmatogenous retinal detachment, regardless of refraction, preoperative laserpexy around retinal breaks, number of retinal breaks and/or uncomplicated cataract surgery. We used the same *exclusion criteria* as Mazinani et al. 2016 and Schaal et al. 2011: previous intravitreal surgery, previous external retinal surgery, previous complicated glaucoma surgery, vitreous haemorrhage inhibiting preoperative retinal examination, age ≤18 years, earlier eye trauma, full‐thickness macular hole, giant breaks, ongoing proliferative vitreoretinopathy (PVR) process, and earlier and ongoing medical diseases affecting the retina.

Exclusions due to complex diagnosis:

• giant retinal tears (4 eyes)
• ongoing PVR process (stage C–D; 36 eyes)
• detachments affected by other retinal diseases; proliferative diabetic retinopathy, full‐thickness macular hole, extensive vitreous haemorrhage and vasoproliferative tumours (10 eyes)
• earlier eye trauma, vitreous surgery or complicated glaucoma surgery (16 eyes)
• Optic pit (1 eye)
• Unclear breaks (1 eye)

Exclusion due to administrative reasons:

• Refusal to give access to medical records (5 eyes)
• Did not return for planned visit (1 eye)
• Lived abroad and was followed up outside of Sweden (1 eye)
• Died before 6 months had elapsed from the day of surgery (1 eye)
• 17 years old at the time of operation (1 eye)
• Endophthalmitis after the primary surgery and secondary retinal detachment (1 eye) *

(* we excluded that eye from the follow‐up of primary and final operational successes, due to the secondary nature of retinal detachment)

Removal of silicone oil (SO) was not considered a failed surgery. Follow‐up of primary and final successes therefore included 230 of the 231 eyes in the entire sample of 228 patients (53 from Örebro University Hospital, 175 from regional hospitals; 144 [63%] men 84 [37%] women; median age 64 years [range 24–94]; median follow‐up time 11 months [range 1–63]).

The operations were performed by 11 different retinal specialists. In some surgeries, a retinal fellow also participated, but there was always an experienced retinal surgeon present. All surgeons made their own decisions about surgical technique, medical treatment and postoperative mobilization. Patients in the university area were called to postoperative examination at the university's outpatient clinic, and those from regional hospitals to those centres. Examining doctors decided when to complete the examination and when to assess the condition as stable. At the final examination, patients were instructed to return if they developed recurrent or new symptoms. The length of follow‐up was decided individually based on baseline status and the course of the disease; therefore, observation times may vary from months to years.

Primary or final anatomic success was defined as the absence of any indication for retina reattachment during follow‐up. Retina reattaching procedures included additional gas injection, second vitrectomy or additional scleral buckling (SB). Indication for any of those was regarded as treatment failure (Goezinne et al. 2010; Mazinani et al. 2016). Additional external retinal laserpexy was not defined as a reattaching procedure, and eyes with oil tamponade were not considered attached until at least 6 months after the SO had been evacuated.

### Surgical technique

Scleral buckling involved a 2.5‐mm encircling band, usually in combination with a custom piece of a 9‐mm biconvex grooved silicon tyre. In most cases, external drainage was performed. Sometimes, intraocular SF_6_ or air was injected. All retinopexy treatments were left to the surgeon's preference. Pars plana vitrectomy (PPV) was performed using the Alcon Accurus vitrectomy system and consisted of a standard 3 or 4 port 20/23 or 25 G vitrectomy using a non‐contact wide‐angle viewing system. Retinopexies of breaks were performed with endolaser probes. The use of heavy perfluorocarbon liquids was optional, and the choice of tamponade was left to the surgeon's preference. Pars plana vitrectomy/SB consisted of PPV as described above combined with an encircling 2.5‐mm silicon band. No other techniques such as pneumatic retinopexy, single buckle or Lincoff balloon were performed. The most common surgical technique was vitrectomy (Table 1).

**Table 1:** Surgical technique.

Surgical technique	*n*	%
Vitrectomy‐laser‐gas	174	75.3
Vitrectomy‐laser‐SO	4	–
Vitrectomy‐laser‐Heavy SO	1	–
Vitrectomy‐encircling band‐laser‐gas	25	10.8
Vitrectomy‐encircling band‐laser‐SO	1	–
Vitrectomy‐encircling band‐laser‐heavy SO	1	–
SB (encircling band and custom piece of 9 mm silicone tyre)	25	10.8
Totals	231	100

### Ethical aspects

This study was conducted according to the declaration of Helsinki. The institutional Review Board/Ethics Committee of Örebro University Hospital approved the design of the study and waived the requirement for informed consent (regional Ethical Review Board [EPN] Uppsala Sweden Dnr:2018/333).

## Results

In total, 206 (89.2%) eyes were vitrectomized. The majority of eyes were phakic 63.1% (130/206). In 19 (14.6%) of these, we operated using a combined procedure involving 18 faco/IOL and 1 faco without IOL due to capsula rupture. Besides these 19 cataract operations, one lensectomy was performed due to increasing preoperative lens opacities. The main ocular tamponade agent used to treat these diseases in vitrectomized eyes was intraocular gas, usually C_2_F_6_ (Table 2).

**Table 2:** Tamponade in vitrectomized eyes.

Tamponade	Eyes (*n*/*N*)	%
C_2_F_6_	181/206	87.9
SF_6_	11/206	5.3
C_3_F_8_	4/206	1.9
SO 1000	5/206	2.4
Heavy SO	2/206	1.0
Unknown gas	3/206	1.5

### Medical records from the university hospital

In total, 228 patients and 231 eyes were operated (53 patients and 54 eyes from the Örebro area.) Most patients who were referred had no postoperative follow‐up notes in our records because they were examined at their home hospital. Our hypothesis was that regional patients who were not re‐referred had presented themselves with an attached retina and no serious complications. If we assume that the hypothesis applies, then primary success = 91.3% (Table 3) and final success = 99.6%.

**Table 3:** Primary success (after first operation) and final success.

Surgical technique	Eyes (*n*)	Primary success (%)	Primary success (*n*)	Failures (*n*)	Final success (%)	Final success (*n*)	Failures (*n*)
Vitrectomy‐laser‐gas	173	90.8	157	16	99.4	172	1
Vitrectomy‐laser‐SO	4	100	4	–	100	4	–
Vitrectomy‐laser‐Heavy SO	1	100	1	–	100	1	–
Vitrectomy‐encircling band‐laser‐gas	25	96.6	24	1	100	25	–
Vitrectomy‐encircling band‐laser‐SO	1	100	1	–	100	1	–
Vitrectomy‐encircling band‐laser‐heavy SO	1	100	1	–	100	–	–
SB	25	88	22	3	100	25	–
Total	230	91.3	210	20	99.6	229	1

### Medical records from the regional hospitals

To review our assumptions, the second step of the study was to compare the postoperative course of patients examined at the regional hospitals with information found in the charts from our surgical unit. Altogether, 175 regional patients and 177 eyes were operated.

Eight eyes from the regional clinics could not be evaluated for administrative reasons stated in the exclusion table. Apart from the eight eyes excluded, as above, all other 169 eyes were reviewed *via* medical record review at least 2 years after the date of surgery.

In this group, we found 16 eyes had re‐detached after primary surgery and one patient suffered from endophthalmitis. All these patients had been re‐referred to the Department of Ophthalmology at Örebro University Hospital for complementary treatment. Apart from these patients, we found no data for any patients who had a re‐detached retina or other serious complication, including endophthalmitis who were not re‐referred, declined re‐referral or were referred to another hospital. Analysing the records from the regional hospitals, we also found that patients we assumed had a successful attachment (as they had not been re‐referred) did have an uncomplicated postoperative follow‐up with attached retina and lack of serious complications. Thus, our hypothesis was confirmed: The outcomes of success and endophthalmitis can be read from the records at the university hospital.

### Reoperations

Of the eyes that did not achieve primary success, 15 were reoperated using *vitrectomy‐laserpexy‐gas*. Of these, 14 eyes were reattached after a total of 25 additional operations. The remaining eye underwent two reoperations, but although the retina was attached centrally, it had a peripheral detachment behind the SO. Due to its poor visual acuity, we aborted further operations on this eye and defined this case as a failure. Only one eye that had a failed primary surgery was successfully attached after one reoperation using *vitrectomy‐encircling band‐gas*, and four others were attached with scleral buckling after a total of six additional operations. Among those eyes included as final successes, we found three eyes with either a small retinal vesicle or a shallow peripheral detachment/retinoschisis in the periphery. These patients were unaware of the findings. We demarcated the alterations with external laserpexy, and they remained stable without any further surgery.

## Discussion

The results of this confirmed our hypothesis to be true and justify the policy of following‐up operative results on chosen indicators by assuming that the charts from the surgical department mirror the whole material. Although our study and those of other researchers differ in many respect, and inclusion and exclusion criteria are not fully consistent across studies, our study shows us that our overall primary and final success rates are well within the ranges found in other international studies (Saw et al. 2006; Schaal et al. 2011; Hajari et al. 2015; Mazinani et al. 2016; Mohamed et al. 2016; Haugstad et al. 2017).

Over the last 20–25 years, SB operations for primary rhegmatogenous retinal detachment have gradually decreased, while primary vitrectomies have increased correspondingly (Schaal et al. 2011; Park et al. 2018). Although many studies have been conducted on various techniques to manage retinal detachment, most are retrospective and focus on differences in success rates between surgical methods. There is currently no consensus on the most appropriate method or methods. Earlier studies found some recurring differences, but it has been difficult to show consistent significant differences between various techniques. Several studies, for example, suggest that SB has a better success rate in patients who are younger, have no PVD and are phakic, which is comparable to, and sometimes even better, than that of PPV (Adelman et al. 2013; Quijano et al. 2016; Park et al. 2017; Park et al. 2018). Scleral buckling is associated with problems such as refractive errors, diplopia, subretinal haemorrhage, hypotension, choroidal detachment and persistent subretinal fluid (Lv et al. 2015; Quijano et al. 2016; Bonfiglio et al. 2018; Park et al. 2018; Fu et al. 2020); however, in younger patients who can still accommodate, and where the appearance of vitreous and retina does not necessitate intraocular intervention, this solution may be preferable. In an uncomplicated retinal detachment with breaks in the inferior part of the retina, SB may even be the best choice (Saw et al. 2006; Quijano et al. 2016; Park et al. 2018).

Our material had only a small subset of eyes operated on with SB, which has gradually declined in favour of vitrectomies internationally and in Sweden. Both primary and final successes for SB in this study are consistent with previous published studies. Although our SB patients were more often phakic than our entire material (84%/56.3%), they have the same median age (64 years).

Pars plana vitrectomy has both advantages and disadvantages. There are obvious benefits in the presence of vitreous pathology, multiple large or central retinal breaks and poor transparency due to peripheral capsule opacities in patients with pseudophakia. Local anaesthesia is usually effective, and the operation and postoperative period are often experienced as less strenuous and uncomplicated than with SB (Mohamed et al. 2016; Park et al. 2018).

The disadvantages of PPV include the development of cataracts in phakic patients, the inconvenience of the gas tamponade and the risk of macular detachment when the gas is resorbed. Iatrogenic ruptures, dislocation of intraocular lenses and unexpected loss of vision and secondary glaucoma are also sometimes seen, especially during and after SO tamponade (Christensen & la Cour 2012; Morphis et al. 2012; Grzybowski et al. 2014; Antoun et al. 2016; Durrani et al. 2017; Wang et al. 2020).

Our material also has a preponderance of C_2_F_6_ use. Many surgeons probably choose the type of gas tamponade based on the number of breaks and their locations, but the choice is also determined by the patient's ability to position or other aspects based on the patient's preference (Sahanne et al. 2017; Guber et al. 2020). We believe surgeons need a special indication to select SO as a tamponade. In addition to its involving an additional operation in connection with evacuation, other obvious risks include PVR development, inconvenience for patients affected in their last eye, the possibility of air transportation (not optional with intraocular gas) or a patient who is unable to position the tamponade. If we decide to use SO tamponade, we should have a clear plan to evacuate the SO within a limited time to reduce the risk of secondary complications (Ichhpujani et al. 2009; Christensen & la Cour 2012; Morphis et al. 2012; Grzybowski et al. 2014; Shalchi et al. 2015; Antoun et al. 2016; Durrani et al. 2017; Wang et al. 2020).

In selecting a type of treatment, one must also consider the risk of complications and the patient's preferences. We prefer to individualize decisions and are not looking for fixed solutions that should apply to all patients.

While we continue to refine our choice and use of current technology, future developments in retinal surgery have already been reported and will surely influence future decisions. Such new developments include endolaser cerclage (Chaturvedi et al. 2014; Falkner‐Radner et al. 2015), standard core and peripheral PPV with air tamponade (Cheng et al. 2020), local dry vitrectomy combined with segmental scleral SB (Fei et al. 2020), glue‐assisted retinopexy in PPV without tamponade (Tyagi & Basu 2019) and localized PPV with air tamponade (Zhang et al. 2017; Bonfiglio et al. 2018).

### Limitations

One of the limitations of this study is its wide‐ranging follow‐up period. Because the study was retrospective, we could not influence the follow‐up time because the physician responsible for each visit determined when the patient's retina could be considered stable. However, one should always be aware that recurrent retinal detachment may be asymptomatic. Although most re‐detachments occur in the first half‐year after the primary operation, the risk of missing re‐detachments increases with shorter follow‐up periods (Goezinne et al. 2010; Lee et al. 2014; Mohamed et al. 2016; Schmidt et al. 2019). To reduce the risk of such misjudgements, patients were asked to return if previous symptoms re‐emerged or new symptoms appeared, and we excluded patients who had died within 6 months of the primary operation (Goezinne et al. 2010; Lee et al. 2014; Mohamed et al. 2016; Schmidt et al. 2019). In addition to monitoring anatomical results, it is equally important to evaluate the development of postoperative vision. This, however, was not possible as the follow‐ups took place at different clinics with different routines around vision examination.

Another limitation could be that the hypothesis is only valid under very special conditions with regard to the national and regional organization of healthcare. We believe that in Sweden, our method may be successful regardless of whether you provide public or private care and regardless of the size of the clinic or the size of society. There are probably opportunities in other countries as well, but it is likely to depend entirely on what policy towards regional clinics and patients the surgical department decides to follow.

## Conclusion

The primary goal of this study was to investigate whether the surgical unit could follow up primary uncomplicated retinal detachment without requesting medical records from the referring clinics. The hypothesis was proven true, which means follow‐up of the results of retinal surgery can be simplified and performed more efficiently. For our clinic, it is important to reflect on why this hypothesis is true. There will always be patients who discontinue their follow‐up, return to their home country before follow‐up, move to another region, die within 6 months after their operation, refuse access to their medical records or refuse further treatment after a recurrence, and in addition, regional hospitals may fail to return that information to the surgical unit.

We believe that close cooperation with all our regional partners is required. Regularly, recurring joint meetings with invited colleagues from all regional clinics to convey new technology, changes in indications and administrative routines have been taking place for a long time and is much appreciated. We also provide for younger colleagues from the regional hospitals to temporarily work at the university hospital for a shorter period of time. Retinal surgeons should also sometimes visit the regional clinics to inform and hold joint discussions together. All contacts between the University Hospital and the Regional Hospital, as well as regional patients, should take place with understanding and helpfulness and an intention of creating a good atmosphere between those involved.

In summary, the eye clinic at Örebro University Hospital has, under current conditions, a professional relationship with our regional clinics that allows us to follow up surgical results of patients with uncomplicated primary retinal detachment based on the medical records available in the university hospital's own medical record system.
